# Implementation of a novel return-to-work approach for persons with affective disorders in a traditional vocational rehabilitation context: a case study

**DOI:** 10.1186/s13033-020-00355-w

**Published:** 2020-03-18

**Authors:** Suzanne Johanson, Urban Markström, Maria E. Larsson, Ulrika Bejerholm

**Affiliations:** 1 Department of Health Sciences/Mental Health, Activity and Participation, Medical Faculty, Lund University, Lund, Sweden; 2 Department of Social Work, Umeå University, Umeå, Sweden; 3 Department of Neuroscience and Physiology, Gothenburg University, Gothenburg, Sweden

**Keywords:** Implementation, Mental healthcare service, Affective disorders, Supported employment, Return to work, Traditional vocational rehabilitation

## Abstract

**Background:**

The person-centred Individual Enabling and Support (IES) model is a novel return-to-work (RTW) intervention for people with affective disorders that was developed from evidence-based supported employment for persons with severe mental illness. Typically, supported employment is integrated into mental healthcare and provides a network around the service user and close collaboration with employment and insurance services and employers. Introducing integrated models into a highly sectored welfare system that includes traditional mental healthcare and vocational rehabilitation is challenging. Greater knowledge is needed to understand how facilitating or hindering factors influence this introduction. The aim of this study was to investigate essential components in implementation of the IES model.

**Methods:**

A case-study was conducted and included four mental healthcare services. Data collection was comprised of semi-structured interviews with 19 key informants, documentation from meetings, and reflection notes. Analyses were performed according to directed content analysis, using the components of the Consolidated Framework of Implementation Research (CFIR) as a guiding tool. Fidelity assessments were performed at 6 and 12 months.

**Results:**

Anticipating RTW support for the target group, and building collaborative relationships and a network with employment specialists that engaged staff in every organization were components that resulted in the greatest facilitation if IES implementation. Barriers consisted of difficulty in integrating employment specialists into the mental healthcare teams, insufficient engagement of first line managers, reorganization and differing perceptions of the IES model fit into a traditional vocational context. Delivery of the IES model had good fidelity.

**Conclusions:**

The IES model can be implemented with good fidelity, several model advantages, and context adaptation. Team integration difficulties and negative perceptions of model fit in a traditional vocational rehabilitation context can be overcome to a certain degree, but this is insufficient for sustainable implementation on a larger scale. Policy and guidelines need to promote integrative and person-centred RTW approaches rather than a segregated stepwise approach. Further implementation studies in the traditional vocational rehabilitation context are needed.

## Background

To assure treatment quality in healthcare, implementation of evidence-based practices into clinical practice is prioritized by Swedish and other national governments and authorities [1, 2]. This priority is also emphasized in the mental healthcare sector [3, 4]. Research on the effectiveness of interventions needs to be combined with implementation studies of how to translate research knowledge into clinical practice. The ultimate goal is benefit for the service users [5]. Many effective and evidence-based healthcare interventions are not successfully implemented and the healthcare sector may not always manage to provide best practices [3]. To achieve positive implementation outcomes, general strategies and critical components in the implementation process must be considered and sufficient resources need to be supplied [6–9]. Studying features of change processes that belong to various levels of healthcare organizations, is suggested to elucidate context specific components that may affect implementation and intervention outcomes [4]. This study focuses on implementation of a recently advanced return-to-work (RTW) model, Individual Enabling and Support (IES), that was developed based on evidence-based processes of supported employment for persons with severe mental illness. Time use, motivational support, and cognitive support strategies were added to better fit the support needs of persons with affective disorders [10, 11].

Challenges in implementation of evidence-based practices could occur during different stages of the process. According to Fixsen et al. [12], thorough planning, education, on-going training, and recruitment of “champions” or experts are important aspects of preparing for implementation. Challenges may also be linked to different levels of the healthcare provision: individual staff, team, and organizational levels as well as communication between levels [13]. One component that could facilitate or hinder implementation is the nature of the new intervention, including its complexity and compatibility to existing organizational values, how the intervention fits within the organizational workflow, and the relative advantages compared to other practices already being delivered [13–15].

In sectored welfare systems, service organizations are administratively autonomous, and collaboration takes place according to regulated agreements. In Sweden, traditional vocational rehabilitation services are spread among welfare organizations. Traditional vocational rehabilitation is defined as services that “help someone with a health problem to stay at, return to, and remain in work”, where *traditional* includes both healthcare and vocational services that are usually provided [16]. The Social Insurance Agency (SIA) coordinates and administers sick leave insurance and the rehabilitation process [17]. Work ability assessments are traditionally performed by medical doctors and vocational training and internship placements are usually provided by the Public Employment Service (PES) when clients are unemployed. Additional sheltered and prevocational rehabilitation is provided by the municipality work administration. Despite regulations of collaboration between stakeholders, differing organizational commitments and responsibilities hinder the RTW processes [18]. In such circumstances, persons with affective disorders are at risk of getting caught in a prevocational loop and not attaining employment [19–22].

In the national guidelines for treatment of persons with affective disorders, the purpose of treatment is stated to be symptom reduction, increased psychosocial functioning, improved quality of life, and return to work or school [23]. However, no RTW intervention is recommended since effective models for achieving employment are lacking. A meta-review on the effectiveness of RTW interventions emphasised individualized support, where a combination of cognitive behavioural and work-place strategies are integrated and provided as an overall solution [24, 25]. The supported employment model contains these ingredients, and IES was shown to be more effective than traditional vocational rehabilitation for RTW [22] and depression and empowerment [26]. In addition, IES costs less than traditional vocational rehabilitation [27]. IES is a person-centred approach and belongs to the vocational rehabilitation paradigm referred to as “place-train” [28], meaning that there is no obligatory work ability assessment or prevocational training before the job searching can start. Instead, the participant is promptly placed and supported in a job according to his or her preferences. This paradigm is based on the recovery and empowerment movement for people with severe mental illness [29] and is in contrast to traditional vocational rehabilitation [28]. Traditional vocational rehabilitation emanates from medical, clinical and juridical perspectives on rehabilitation and is focused on a decrease of symptoms and prevocational rehabilitation with work ability assessments known as “train-place” [28]. Earlier implementation studies show that integrated vocational models such as IES, that needs closer integration of healthcare and other authorities than in traditional vocational rehabilitation, can be difficult to implement in sectored welfare systems with contrasting logic and regulations [7, 30, 31]. Because of the contrast and potential conflict between the two approaches, understanding the implementation challenges of IES (“place-train”) when implemented in a traditional vocational rehabilitation context (“train-place”) is essential.

### Aim

The purpose of this study was to investigate important components in implementation of the IES model, and how these components influence implementation. The CFIR was used as an analytical tool, to illuminate potential facilitators and hindrances when implementing this novel RTW approach in a Swedish traditional vocational rehabilitation context.

## Methods

The following questions guided the study:

1. Which key components of CFIR can be identified in the implementation of IES?
2. How are the identified components related to each other in terms of enhancement or detraction of implementation?
3. What significance do the components have for IES implementation in a traditional vocational rehabilitation context?

An embedded case-study design was used to obtain a deeper understanding of barriers and facilitators in IES implementation [32]. When the context is important, the case-study is a suitable method because it interacts with the studied phenomenon [32]. The implementation process was bounded in time between 2011 and 2014. IES implementation was performed in the context of the mental healthcare organization and other welfare organizations. The Consolidated Framework of Implementation Research (CFIR) by Damschroder et al. [13] was used to guide data collection and data analysis. CFIR introduces a meta-theoretical frame of reference compiled from implementation research and offers key concepts for evaluating an implementation processes, includes several implementation levels, and internal and external organizational components [13]. The framework is composed of five domains and each has several components. On a comprehensive level, these domains can be viewed as interacting and impacting each other and the components [13]. The five domains are intervention characteristics (e.g., relative advantage, adaptability), outer setting (e.g., patient needs and resources, external networking/collaboration), inner setting (e.g., networks and communications, implementation climate, perceived meaning of intervention), characteristics of involved individuals (e.g., interplay between individuals and organization), and implementation process (e.g., planning, engaging opinion leaders, executing) [13]. Opinion leaders can be staff members who can influence their colleagues about attitudes and perceptions of a new intervention.

### Case setting

The IES project was part of a national research venture regarding vocational rehabilitation interventions for people with affective disorders and/or long-term pain (REHSAM), with the purpose of evaluating interventions that could lead to decreased sick leave and enhanced opportunities for people to RTW. The IES intervention was organized through a REHSAM steering committee in which project leaders, head or strategic healthcare planners, SIA and PES participated. The IES context and case setting consisted of four distinct mental healthcare units for people with affective disorders (out-patient units), but was evaluated as one implementation process because it was setup as a coherent project. The four units were selected by the steering committee and located in geographically diverse sites in southern Sweden. The units provided one or two multidisciplinary teams responsible for people with affective disorders and covered 16 municipalities. Medical doctors, nurses, psychologists, occupational therapists, social workers and physiotherapists provided regular treatment and medical rehabilitation at each unit. Every mental healthcare unit had a first line manager, a joint head of department, and moreover, chief medical officer of mental healthcare services in the county council.

The SIA and PES heads from the southern district in Sweden were informed about and agreed with the project. The first line SIA and PES office managers became involved. Two employment specialists were recruited for the project. Their role was to undertake the RTW support according to IES, and each employment specialist covered two mental healthcare units. The IES was organised according to the Supported Employment Fidelity Scale (SEFS) [33], meaning that each participant was recruited from a team in the mental healthcare unit and each ES served specific teams. The steering committee and integration of IES with SIA, PES and employers was part of the fidelity.

### The IES model

The IES model is based on evidence-based supported-employment principles that were originally designed for persons with severe mental illness. An important function of the support is to build a network around the participant where coordinated support is provided by the employment specialist and team members from mental healthcare, in collaboration with SIA and PES handling officers. The employment specialist takes the lead in RTW support and is guided by the IES principles. The IES enabling component introduces support by mobilizing motivational, cognitive, and time-use strategies. The purpose of these strategies is to give users the opportunity to handle change in their process towards work and to develop coping strategies for work engagement. Time-use strategies are used for balancing other daily activities and to promote a working life. The IES model is described in detail in a previous study [10]. The enabling component is then integrated with the supported employment principles, which include competitive employment as the goal, eligibility based on the person’s willingness to participate and work, job searches that should start early in the process, support based on the participant’s preferences, integration of the vocational plan with mental healthcare treatment, continuous and not time-limited support, benefit counselling, continuous job recruitment, and employer networking [34].

### Selection of informants

Interviews were performed with purposefully selected key informants [35] from the different organizations involved in the project. We targeted informants who were involved in decisions about the implementation and performance of IES. Exceptions were made because of staff turnover during the study; one mental healthcare manager and two opinion leaders changed work assignments, and two first line managers from the PES offices finished their duties. Other inclusion criteria related to informants at different organizational levels and settings. Thus, employment specialists, chief medical officers of mental healthcare services, first line mental healthcare managers, mental healthcare staff including opinion leaders, strategic planners from the county council, first line managers, and handling officers from local SIA and PES offices were selected. Informed consent was collected from each participant and all procedures were in accordance with the ethical standards of the responsible committee on human experimentation and with the Helsinki Declaration of 1975, as revised in 1983. The study was approved by the Regional Ethical Review Board at Lund University (Dnr 2011-544).

### Data collection and procedure

#### Fidelity

High fidelity score is associated with positive RTW intervention outcomes [36] and implementation outcomes [9]. Fidelity was first assessed according to the Supported Employment Fidelity Scale (SEFS) [33] at 6 and 12 months by one of the researchers (UB) who is educated in fidelity assessment according to international standards. The assessment was then validated in a consensus meeting to adjust for assessment bias. Two additional persons, a researcher (SJ) and a project member, attended the consensus meeting. The idea was to assess the degree to which the IES intervention was implemented as intended. The SEFS scale consists of 25 questions comprising areas of staff, organization, and service. Three questions about motivational, cognitive, and time-use support were added to cover the enabling component of the IES service. Criteria for collecting data and assessing items are described in detail in the SEFS manual. For example, materials from employment specialists, head of mental healthcare units, and vocational log documentation were used and triangulated. Each item was rated on a 5-point scale and fidelity sum scores range between 25 and 125 points [33]. Less than 73 points equals ‘not supported employment’, 74–99 points is fair fidelity, 100–114 points is good fidelity, and 115–125 points reflect exemplary fidelity. The IES-specific items of enabling strategies add up to 15 points, where 0–4 corresponds to low fidelity, 5–9 to good fidelity, and 10–15 to exemplary fidelity.

### Interviews and text materials

Text materials from meetings and reflection notes from the planning, preparation and performance of the project, and semi-structured interviews were collected. Several data sources provided multiple perspectives in the analysis. Nineteen interviews with key informants were conducted during the last 6 months of the project. Each interview lasted between 40 and 75 min, was digitally recorded, and transcribed verbatim. The interview guide was constructed with guidance from CFIR. The wording was adapted to fit groups of key informants belonging to specific organizations. The first questions were open-ended, then probing questions were used to investigate the key informant views of the implementation.

### Data analysis

Fidelity ratings by SEFS items were summarized into a total score. Interviews and text material were analysed using a directed content analysis where codes and categories followed the concepts of CFIR domains and components [37]. The first author (SJ) initiated the coding process with the crude material, and all co-authors reviewed and adjusted the coding in an iterative process. To further validate emerging findings, the last author (UB) reviewed the analysis process. A summary of the implementation process was then created and described in chronological order. In the CFIR framework, implementation process is the last domain. However, to maintain chronological order, the implementation process is presented as the first domain in the findings.

To add detail to the analysis of the implementation process, the four embedded mental healthcare units were analysed in two steps. First, a synthesis of important components of CFIR for each unit was made, and then a comparison of the units was done with respect to component value (see Table 1). This was achieved by applying an overall rating approach as inspired by Damschroder [38]. The rating consisted of two criteria, with either a positive or a negative response to the implementation. To discriminate between a predominantly positive or negative influence, concrete examples and explicit descriptions were derived from the interview material and text.

**Table 1:** Summary of components that facilitated or hindered the implementation process according to interviews and documentation

Facilitating components	
Process	Planning: Meetings and dialogues about model fit involved all organizational levels Engaging: Successful recruitment of opinion leaders Executing: Continuous meetings with opinion leaders, distribution of newsletters, supportive feedback
Intervention characteristics	Relative advantage: Appropriate support for the target group; people with affective disorders are in need of RTW support Employment specialist competence of labour market and psychiatry Person-centred, continuous and not time-limited support
Inner setting	Networks and communication: Employment specialists built constructive relationships and functioning teams with engaged staff members in the mental healthcare units Opinion leaders enhanced collaboration at the mental healthcare units, and this was important for the ongoing intervention
Outer setting	Cosmopolitanism: Fruitful collaboration was developed in some PES and SIA services
Hindering components	
Process	Planning: Large geographic area and involvement of many organizations made implementation complex Major reorganizations delayed implementation Engaging: Difficulty engaging opinion leader in one mental healthcare unit Lack of time and work overload for first line managers
Intervention characteristics	Relative disadvantage: Staff members in mental healthcare units had divergent opinions about the IES model fit into their organization Informants from the PES modestly questioned the IES model advantage
Inner setting	Implementation climate: Difficulty for employment specialist to integrate into existing mental healthcare teams Compatibility: Responsibility, commission, and financing of RTW support was perceived as unclear, related to vague guidelines for vocational services and organizational boundaries
Outer setting	Cosmopolitanism: Collaboration with PES and their subcontractors was complex and time-consuming Patient needs and resources: Differing perspectives on how to design RTW support, location of internships and vocational training were proposed by the PES according to regulations

The CFIR does not include implementation outcomes. However, Damschroder et al. [38] show how to operationalize important outcome measures for a separate project. In the present study, findings are discussed in relation to fidelity and according to the implementation outcomes: *acceptability*, *adoption*, *appropriateness,* and *feasibility* as suggested by Proctor et al. [39]. These are possible outcomes in an early implementation stage.

## Results

### Fidelity

Fidelity rating was performed on the IES delivery. At 6 months, the sum score was 101 points out of 125, and there was 12 points out of 15, for the IES-specific features of motivational, cognitive and time-use strategies. At 12 months, there was an increase to 106 points out of 125, and an improvement to 14 points out of 15 for the enabling strategies. Both assessments corresponded to good fidelity. The items that were rated high [5], related to employment specialist engagement in all phases of the IES intervention, sufficient caseloads, a focus on regular employment, zero exclusion, benefit guidance, support from employment service, disclosure (or not) of mental illness to employer, individualized job development, different kinds of jobs and employers, and ongoing and outreach support according to experienced needs. A rating of 4 points was for a stable employment specialist working group, a steering committee, continuous assessments of profiles and plans, rapid job placement, and a community-based service. The integration with mental healthcare teams and establishing relationships with employers were each rated at 3 points. Supervision was scored at 2 points, and permanent employment over 90% was a rating of 1 point.

Score changes between 6 and 12 months corresponded to time employment specialists spent exclusively on RTW issues (5 to 4 points), supervision (3 to 2), establishing relationship with employers and integration with team (2 to 3), and individualized job development (3 to 4).

### Identified components in the IES implementation

Both facilitating and hindering CFIR components were identified in the interview and text material. The components mainly belonged to the domains of intervention characteristics, outer and inner setting, and process of implementation. No components from the domain of characteristics of individuals were found.

Identified barriers were mostly associated with planning and engaging (e.g. difficulties in engaging opinion leaders from the start), implementation climate (e.g., difficulties in integrating the employment specialists into the mental healthcare teams), compatibility or model fit, and challenges in patient needs and resources and cosmopolitanism. The findings are presented below according to CFIR domains, starting with the implementation process. A comparison between the mental healthcare units is outlined for each domain and in relation to hindering and facilitating aspects of implementation.

### Implementation process

#### Planning

Initially, a steering group and IES staff group were planned and organized. The steering group decided that the IES should be implemented in mental healthcare context and not in primary healthcare, as was planned in the project application. Four mental healthcare services and their teams were chosen based on having no other ongoing project. The head of the mental healthcare research department contacted the units, and then, together with the principal investigator, met one at a time with the first line manager of each unit. At this point, IES communication material was designed and delivered to introduce the IES to the staff. Next, staff meetings were held to inform staff about the IES model and the importance of integrating the team into RTW support for employment success. IES was marketed as an important opportunity for the patients who wanted to RTW. In parallel, an IES communication guide was developed and used in dialogues about intervention fit, with the SIA and PES heads in southern Sweden.

Differing opinions emerged regarding intervention fit, even though there was a consensus of the need for RTW support for people with affective disorders. Although implementation attitudes were mostly positive during the planning meetings, none of the first line managers had the time to genuinely engage. Work overload was described by the manager in unit 1, where basic functions such as having consistently employed psychiatrists was a challenge. External factors, such as major reorganizations in unit 1 and 2, negatively impacted the planning phase. First line managers and staff who were engaged initially moved to other units or teams and needed to be replaced. At these units there was an overall perception that the staff was overloaded and worn-out due to the reorganizations. One manager in unit 2 seriously considered declining participation in the IES intervention, but remained involved. The reorganizations in the first two units prolonged the planning phase, which impacted the other units. In units 3 and 4, the planning phase was experienced as too long and complicated, and this influenced attitudes toward the intervention among the staff.

Similarly, meetings with first line PES and SIA managers revealed somewhat different perspectives as to whether IES would fit into the current welfare system and RTW support. Three first line PES managers agreed to the project, and one expressed disinterest. This negatively impacted local PES collaboration at that site (unit 4), throughout the project. All three SIA line managers approved of the IES implementation. The SIA and PES staff were informed at each local office.

#### Engaging

The next step was to engage opinion leaders among the mental healthcare staff that were responsible for moving information in and out of the IES and healthcare organization. They also took responsibility for running the practical implementation work at each service unit, and spread positive attitudes about the intervention to their colleagues at team meetings. Opinion leaders were successfully recruited at units 1–3, but not unit 4 since the appointed opinion leader moved to another service. Engaging another staff member as an opinion leader at unit 4 was difficult. Despite repeated meetings with the first line manager and staff, unit 4 had reservations about the intervention and no openings for adaptations of work routines, which made implementation complicated. In contrast, unit 3 engaged several of their staff members in addition to the opinion leader.

During the first year, the project coordinator (SJ) met with the opinion leaders every third week to facilitate the process, and regular newsletters with information about intervention progress were distributed to all levels within the IES organization. Execution of implementation steps at unit 4 when the project was running, such as continuous support and feedback, was not accomplished because of general disinterest. The opinion leaders at units 1–3 further enhanced collaboration and supported the implementation by engaging in the process development. They conveyed that there was a current lack of RTW support for the target group, and promoted the IES intervention to other staff members as a suitable model.

### Intervention characteristics

#### Relative advantage

There was a general perception in the mental healthcare units that RTW support is needed for the target group of people with affective disorders, and the model was largely perceived as advantageous. A person who provides individual support (the employment specialist) was perceived as being appropriate to participants’ needs, particularly because that person had competence in both the labour market and psychiatry. The first line managers and opinion leaders also underscored the importance of a model that builds a bridge between mental healthcare and traditional vocational rehabilitation. The individual focus and providing a model where work support is not limited in time and space were also portrayed as benefits.

*Right now, the IES is introduced in a natural way in our service, and most of the staff have a positive attitude. Otherwise, we discuss the need of connecting to the vocational service when we assess a patient to be ready for that, but often some kind of barrier turns up…there really is no bridge between healthcare and vocational service although there is a need for that. My impression is that the IES model has a good potential to fit in there. (Opinion leader at a mental healthcare unit)*

All informants but one from the PES agreed with the IES model. They also expressed a general need to improve their knowledge within their own organization regarding mental disorders. Although they could see model advantages, they presented a more modest attitude towards the IES as they had provided supported employment for people with physical disabilities for a long period of time, and therefore assessed the IES to be a similar model. In this sense, appropriateness for IES was thought to be low in three of the employment offices. Only individual managers and case workers at some of the offices adopted the intervention.

*Supported employment is principally the same as our employment advisors…These advisors meet participants who are readily assessed regarding work ability, all assessments are finished…by then we know how much they can work, if adaptations are necessary, and so forth. They need to be job*-*ready, so to speak, to receive support from an employment advisor. (PES Caseworker)*

### Inner setting

#### Compatibility

The key informants were sceptical about work achievement for persons with mental disorders because of labour market structures. They were convinced of the necessity of providing RTW support for persons with affective disorders, and that was a strong argument for them to become engaged. They reflected upon vague policy and guidelines regarding vocational rehabilitation, and the different assignments that mental healthcare and PES have, but that did not hinder engagement. In contrast, the first line manager for unit 4 felt that the intervention did not fit into the mental healthcare organization. Instead, that manager was of the opinion that treatment and care were the main functions of mental healthcare, and vocational rehabilitation was an assignment for other authorities. The first line managers in unit 1 and 3 also referred to unclear treatment guidelines for affective disorders, but were more modest in their questioning of who was responsible of vocational rehabilitation. Thus, indistinct guidelines could complicate staff understanding of the mental healthcare service mandate.

*According to me, the guidelines are vague, and I think our mission is not clear…for the co*-*workers in mental healthcare it is not clear what they are supposed to do concerning the service*-*users’ vocational rehabilitation… and I do not think they have knowledge about what kind of alternatives there are in the municipality, the social insurance agency or the employment services…it is a challenge to stay up to date on authorities’ various support. (Chief medical officer of psychiatry)*

A discussion of where the employment specialists should be employed and work emerged from the key informants of mental healthcare service. They suggested joint employment where both the mental healthcare services and the welfare authorities should take responsibility. Financing of medical and vocational rehabilitation was explicitly described as an issue by key informants of the county council on the strategic level. Among mental healthcare staff, specialised medical competence, rather than having advanced knowledge of RTW support, was highlighted as essential for quality treatment in the mental healthcare service mission.

*What is the mission of mental healthcare service? That question is very important, I think. People have told us that mental healthcare is dedicated to treatment, not rehabilitation. We have had discussions about this with the staff and they have questioned if it is their role to rehabilitate…. The mental healthcare service has been like a sealed fortress, where only some interested opinion leaders have opened the door. (Employment specialist)*

#### Networks and communication

A statutory organization of joint funding of rehabilitation projects (Finsam) that incorporates the mental healthcare, PES, SIA and municipality is regulated to enhance authority collaboration. This was emphasised by the first line mental healthcare managers in unit 2 and 3 to be an important organizational structure for cooperation. Since IES was not part of this cooperation, but was implemented as a research project, it did not initially have the same legitimacy as other ongoing projects at unit 3. However, when IES participants started to announce their positive experiences with the RTW support to the mental healthcare staff, legitimacy of the model increased. Most of the staff did not address work in their meetings with the service user. They perceived this area as being the responsibility of PES. Opinion leaders in mental healthcare opposed this perception and underlined the need for close teamwork with the service user when planning for RTW. From the employment specialists’ point of view, it was obvious that there was no tradition of a team or network around the service user with whom they could collaborate on a regular basis. Some units had team meetings for information sharing about service users, but there was no structure for internal collaboration where, for example, RTW issues could be addressed. In addition, having the employment specialists attending the teams was questioned by several staff members because of confidential information that would be discussed. The employment specialists felt that they were welcome in the mental healthcare service, but at the same time were perceived as a resource that did not belong to the mental healthcare team. This was perceived as the main barrier for the implementation process.

*I sometimes experienced that the case managers did not want to disclose service user information, they kind of kept secrets in the team… although I always showed the informed consent from the participant. There was an opposition in a way…it was not easy to become approved of in the team. Not like “welcome to the team, let’s work together!” (Employment specialist)*

To get around this problem, the employment specialists developed collaboration with the staff or case manager who met most regularly with the participant, likely a physiotherapist, psychologist, social worker or occupational therapist, and sometimes a psychiatrist. This created a resource team around the participant in accordance with IES principles. However, sometimes staff finished their treatment when participants met the employment specialist. In units 1 and 4, networking difficulties impacted the implementation throughout the process, whereas the first line manager at unit 3 corroborated the lack of team work, and expressed the need for more internal communication between staff professionals to facilitate a holistic approach in support and treatment. Most staff at unit 2 perceived the employment specialist as an external resource, who did well for the service user, but their own engagement was not perceived to be needed for that support. Overall, collaboration was difficult with the psychiatrists because of a lack of their time, staff turnover, and the use of interim “rental” doctors.

### Outer setting

#### Patient needs and resources

Inter-organizational communication and collaboration was reported to negatively influence the implementation process. One reason seemed to be differing perspectives of how to design the RTW support. Several participants needed to prove that they had resources and to start internships and vocational training before searching for regular employment, consistent with the current regulation and agreements of traditional vocational rehabilitation. This was in contradiction to the IES model that promotes rapid job development based on the preferences and resources of the participant. However, some handling officers at the local PES offices, adapted to the place-train RTW process because they were convinced of the benefits for participants to follow their own plans and find employment first. Others referred to regulation agreements and provided no opportunity for adaptations. From the start, two first line PES managers were determined of that the IES intervention would fit well into their new rehabilitation approach. However, as the IES implementation started, those two managers moved to other jobs. The first line managers who replaced them did not engage in IES and consequently collaboration was curtailed. Another collaboration challenge was with PES use of subcontractors for vocational training and internships. This made collaboration even more difficult due to many actors, and impacted the integrated RTW approach of IES.

#### Cosmopolitanism

One employment specialist could develop close and constructive collaboration because of the enthusiasm of a first line manager in a local PES office. By finding local solutions to administrative regulations, there was an opportunity to follow participants’ preferences and needs as is consistent with the IES model. Another handling officer, who was responsible for rehabilitation at the PES, understood the IES intervention to be a service complementary to their organization. Because the IES project was not subcontracted by the PES, regulations prevented it from being requested.

*Some handling officers gave us more independence to search for jobs immediately, and then evaluate. Others more strictly followed the traditional rehabilitation where vocational training and internship are the first step. As an employment specialist, I then needed to adapt my role in relation to the individual support and tried to hasten the process as much as I could. The collaboration was smoother if the participant belonged to the so*-*called “work introduction phase” within the PES. (Employment specialist)*

The employment specialists needed to adapt to regulations to a great degree and this impeded implementation. Although the employment specialist and participant constructed a career plan and job profile together, handling officers wanted to do their own work ability assessment, and refer to internships that the participant could not choose by themselves. There was also an embedded problem regarding norms and values of work ability of people with affective disorders, which limited opportunities to achieve regular employment without subsidies. Even though PES informants stated that it was important to aim for regular employment, they perceived that people with affective disorders need support to undertake work, and therefore there was an obligation to compensate the employers financially. This tool and employment measure are regularly used in employment processes and agreements with employers, but it is not entirely in line with supported employment principles. In retrospect, some participants started employment with subsidies because of the regulations, but later achieved regular, unsubsidised employment.

Collaboration with the SIA mostly worked well, but was sometimes aggravated by bureaucracy. For instance, administrative difficulties occurred when long-term economic benefits needed to change because participants achieved employment. This lengthened the process. However, overall there was a general interest in supporting the participants. The SIA handling officers often shared the same values as in supported employment, which facilitated the collaboration between them and the employment specialists.

## Discussion

The implementation process of a newly advanced RTW approach in a traditional vocational rehabilitation context was studied. The study was framed and time-limited as a project, and the findings need to be viewed in this light. The focus on implementation outcomes at the beginning and during the process resulted from the time limitations, and sustainability of the implementation has not been evaluated. The perceived relative advantage was that RTW support was perceived as being needed for the target group and could bridge the gap between mental healthcare and vocational services, and this facilitated acceptance of the model. Implementation was facilitated when employment specialists could build important relationships and collaborations with dedicated staff in mental healthcare, SIA, and PES in line with IES principles. Reaching adherence to evidence-based practices of supported employment is possible, as shown by the enabling strategies and fidelity results at 6 and 12 months. Approximately 40% of the participants in this context achieved regular employment after 12 months, and only 4% of those who received traditional vocational rehabilitation did so [22]. In addition, those in IES became less depressed and more empowered [26]. The components that had the most influence on the implementation process are discussed below.

According to Meyers et al. [8], a successful implementation needs to begin with several steps of need, readiness assessments, and careful planning before the actual intervention begins. The current study achieved this by conducting several meetings at all organizational levels and included the question of intervention fit into mental healthcare and traditional vocational rehabilitation contexts. The primary response from chief medical officers and three of the four mental healthcare managers was a positive attitude toward the IES model. First line managers at the PES offices had differing perceptions of the model advantages. An implementation process can be delayed or prevented if there is lack of acceptance or adoption of the new intervention, and this further impacts feasibility [4]. The first line managers and staff who had a resistant attitude to the IES model in the beginning of the implementation process, did not accept the model and did not adopt it, but they were the minority. The fact that most managers and staff claimed the importance of RTW support for people with affective disorders opened the door for the implementation. Networking with engaged and dedicated staff was shown to be crucial. The employment specialists built their own functioning teams which is consistent with the evidence-based practices of supported employment as reflected in the fidelity assessment. Part of the complexity in healthcare interventions relates to various professionals who are obliged to collaborate both within and outside their own context [5]. Sound networking and building sustainable relationships are shown to positively impact implementation [13], and this was the key to successful RTW support in our study.

Reasons for resistant attitudes to a new intervention can be many. Providing RTW support in mental healthcare was questioned because the treatment guidelines for affective disorders were perceived as mostly supporting treatment directives. The guideline directives were generally perceived as ambiguous, and this made their interpretation more difficult. This led to ambivalence about medical and vocational assignments. Most of the mental healthcare managers and staff members were anchored in the medical perspective, and medical skills were given priority over RTW knowledge. On this basis, and because of work overload, work issues were not addressed in the teams, and it was challenging for the employment specialists to become involved in the traditional team work of sharing clinical information. Healthcare is provided in hierarchical structures where front-line staff are dependent on guideline directives to complete services according to regulations. If the guidelines are perceived to mostly address a medical perspective, this will be how staff members respond [40, 41]. To achieve a change in the mental healthcare directives to include and actively integrate RTW issues, additional randomised trials will be needed to establish an evidence-based practice, and policy guidelines will need to be changed. For example, the governments in Norway, Denmark and Great Britain integrate the policies for work and mental health, and promote evidence-based practices of supported employment on the national level [42–44]. Policy change might also solve some of the collaboration challenges between mental healthcare and other welfare organizations in RTW. Organizational culture and individual behavioural change are reported to be other important factors in implementation research [13, 45–47]. New interventions demand behavioural change from staff members, and hindrances may result when there is resistance to the new model [40, 41, 46]. Damschroder et al. [13] discuss the construct of implementation climate, and refer to receptiveness of new interventions, perceptions of the need for change, and whether change is important to involved individuals. The relative advantage of the IES model, and subsequent acceptance of the model made several staff members prepared to work according to new routines. This confirms previous research on individual behavioural change [41].

According to Damschroder et al. it is easier to implement a model when there is open communication between staff members, when staff feel connected to each other, and there is a common goal. Leadership is an important implementation factor because managers influence staff members, as well as what is done and achieved in the organization [13, 47]. Insufficient leadership engagement was illustrated in one of the units, but this was partly compensated for by the perception of the IES advantages.

### Methodological considerations

This study used an embedded case-study design [32], and the CFIR framework was applied to investigate important components when implementing the IES model in a traditional vocational rehabilitation context. The case-study design was useful since the implementation was performed in a complex context and was time-limited [32]. The embedded units were four mental healthcare services that were evaluated as one uniform process. To strengthen study trustworthiness, the implementation was thoroughly planned and prepared for in advance, and was followed to ascertain that each step was performed in the same way in the mental healthcare unit, PES, and SIA contexts. Although there were enough mental healthcare units and teams involved according to fidelity, too many actors had to be contacted when integrating the IES. The employment specialists had to spend an excessive amount of time on transportation when visiting the different units and related settings. Thus, too many stakeholders and too much administration impacted implementation. Future implementation research on IES would benefit from choosing a context where collaboration and integration of services is possible.

Diversity and quality in data material was achieved by collecting fidelity ratings, notes from planning meetings, when decisions were made during the implementation process, meeting protocols for each meeting with heads, first line managers and staff members, and interview material from 19 key informants. However, by not including participants of the IES intervention as key informants, might limit this study, as their perspective of various implementation factors is lacking. Implementation fidelity was assessed with the SEFS measurement scale, which is standardized in relation to evidence-based supported employment principles [33]. Three additional questions about the enabling components also added to the fidelity assessment. These questions followed the same construction logic as the SEFS questions, and a description accompanying each rating level [1–5] was developed and described by experts of motivational, cognitive and time-use strategies. The questions in the semi-structured interview guidelines were based on a theoretical framework for implementation (CFIR), which adds to the study’s transferability [48]. Because domains and components in CFIR comprise important implementation factors that have been researched in various contexts, the concepts are likely transferable [7, 38]. The framework enhanced interpretation of how the components are related to each other. In the directed content analysis, the framework guided the analysis. This further strengthened the credibility as CFIR provided predefined concepts. The analysis was conducted in an iterative process by all authors to further strengthen the trustworthiness. Credibility was strived for by using quotations in the text.

## Conclusions

The IES model can be implemented in the traditional vocational rehabilitation context, as reflected in the fidelity scores. Taken together, several aspects of the relative advantages of the IES model and consensus of the need for RTW support for the target group enhanced acceptability of the model, and this played a crucial role in starting and facilitating the implementation process. Informants perceived the IES model to fill the gap between mental healthcare and vocational services, which indicates that there might be a need for integrated RTW support. However, there were difficulties in integrating IES within the existing mental healthcare team structure, and scepticism of model fit into the workflow of traditional vocational rehabilitation made the implementation process longer and more complicated [30, 31]. Further studies on implementation of IES into the traditional vocational rehabilitation context that include policy guidelines and sustainability perspectives are warranted.

## Data Availability

According to Swedish Ethical Review Authority it is not possible to publicly publish datasets used in this scientific study. However, datasets used in the current study are available from the corresponding author on reasonable request.

## Abbreviations

IES: Individual Enabling and Support model
RTW: Return-to-work
CFIR: Consolidated Framework of Implementation Research
SIA: Social Insurance Agency
PES: Public Employment Service
REHSAM: Rehabilitation and Collaboration
SEFS: Supported Employment Fidelity Scale
